# Culture, humanities, evolution: the complexity of meaning-making over time

**DOI:** 10.1098/rstb.2020.0043

**Published:** 2021-07-05

**Authors:** Joep Leerssen

**Affiliations:** Professor of Modern European Literature, University of Amsterdam, Kloveniersburgwal 48, 1012 CX Amsterdam, Noord-Holland, The Netherlands

**Keywords:** culture, humanities, history, emic and etic, autopoiesis, complexity

## Abstract

This article outlines how the historical human sciences see ‘culture’ and its dynamic developments over time and over generations. The operations of human culture are systemically self-reflexive and, as a result, exhibit a complexity that sets them apart, as a semiotic system, from mere communicative information transfer. Peculiar to this complexity is the two-way interaction between the ‘etic’ substance of the cultural exchanges and their ‘emic’ function. Cultural signals require parallel etic/emic processing at stacked levels of complexity. As a result of this complexity, the homeostasis and autopoiesis of human culture, including its dynamics and development over time, cannot be explained fully in terms of responses to the physical environment. How, this article ponders by way of conclusion, can an evolutionary approach be reconciled with these characteristics of human culture, or the notion of culture be applied to evolutionary modelling?

This article is part of the theme issue ‘Foundations of cultural evolution’.

Being aware of being aware of being. In other words, if I not only know that I am, but also know that I know it, then I belong to the human species. All the rest follows.

([1], p. 142)

## 1. Introduction

The application of ‘culture’ to ‘evolution’ (or vice versa) covers widely divergent scientific debates and practices [2,3]. Underlying these is a fundamental crux: what does either word mean in the light of the other? The present paper aims to bring to this question some insights from the long reflection on culture within the humanities—those sciences that have been analysing cultural artefacts and cultural production ever since Aristotle's Poetics. ‘Cultural History’ as a specialism within the historical sciences goes back to Jakob Burckhardt's classic *Die Kultur der Renaissance in Italian* (1860), drawing on still older scholarly traditions: literary history and art history. It is still thriving today [4–6]. Its tenets and insights have developed in interdisciplinary contacts with anthropologists^1^; anthropological theory, for its part, has been drawn upon by primatologists, palaeontologists and biologists; but I find little evidence of a direct dialogue between the historical humanities and the bio-sciences or empirical sciences about the mutual applicability of ‘culture’ and ‘evolution’. Reflections on culture have, for centuries, been the core business, the very definition, of the humanities; so central and fundamental, perhaps, that meta-reflections on the meaning of that concept were left to philosophers, together with the meaning of beauty, of humanity or of life itself. It took encounters with the rising empirical sciences to force the humanities to look into that mirror. Fifty years ago, the poet and Nobel laureate T.S. Eliot felt it necessary to publish his *Notes towards the Definition of Culture* [9] because the term had been operationalized in the mission of the recently established UNESCO, furnishing the C in that acronym. Shortly afterwards, C.P. Snow published his epochal *The Two Cultures* [10] on the incompatibility between the human and the natural sciences. A half-century later, the encounter with Cultural Evolution concepts in the bio-sciences and empirical sciences provides a welcome opportunity to update those reflections, and to explain to others and to ourselves what has been intuited as ‘culture’ in the cultural sciences since Aristotle and Giambattista Vico.

As a cultural historian, I admit this with some shame. ‘Culture’, fundamental as that concept is for the humanities, has not been given a generally accepted definition or operationalization in the human sciences and has been loosely applied—even by critics like Eliot, who does little to actually deliver the definition promised in his title. (Much more was achieved by sociologists and anthropologists, on whom I shall rely extensively in the following pages.) Given the failure of the humanities to provide anything like a conceptual benchmark, is it any wonder that the empirical sciences should use humanist or culture-related concepts in any other than an informal, often metaphorical way? Those metaphors bespeak a certain tendency to downplay the specificity of humanity and culture in its relations to the mechanical operations of the natural world. In certain publications, human subjects tend to be rhetorically reduced to their mere physiological stimulus–response operations^2^; conversely, phenomena of a statistical, mechanical or chemical nature are metaphorically humanized in terms of their inner volition. Besides the colloquial characterization of market forces as ‘nervous’ or ‘confidential’, there is the imputed ‘selfishness' of Dawkins's genes, which are—metaphorically, of course—credited with a sentience and intentionality that is—metaphorically, of course—denied their human hosts. The language is playful, ironically coquettish, but also heedless of the imprecision it celebrates. And the humanities only have themselves to blame for this, because how we use language, and how the experiential human Self shapes its interaction with the world by means of Culture: all that is the humanities' remit, and they have failed to give any basic explanation in the matter.

This article raises the problem how assumptions on culture, fundamental as they are in the long-established field of the humanities, and commonsensically informing our non-technical language usage and self-understanding as humans, can be validly applied to the scientific praxis (or practices) of analysis in the thriving field self-defining as ‘Cultural Evolution’. In what follows, I will make explicit the idea of culture and its connotations (also as regards its dynamics and development) as informing the humanities as I see them (§2). I then make an attempt at putting that understanding of culture in discipline-neutral terms. I rely on complexity theory to present culture in terms of its recursive self-reflexivity (§3); and I rely on the well-established anthropological distinction between culture's etic elements and emic functions in order to sharpen our focus on the operations of that systemic recursivity (§§4 and 5). This, I hope, will clear a space in which we can establish, in an interdisciplinary discussion of pitfalls and possibilities, what is involved in studying culture in evolutionary terms, or operationalizing the cultural aspects of evolution.

## 2. How things appear to a historian

The human reflection on culture is part of human culture. In this opening section I point out that such meta-cultural reflections traditionally situate human pursuits in a heuristic opposition between ‘culture’ and ‘nature’. This opposition is analogous to the distinction between ‘humanities’ and ‘natural sciences’. In raising the question of evolution as an operative principle, this first section establishes that for most of the human sciences the understanding of historical variability (diachrony) is a central concern, but that this diachrony, based as it is on the availability of textual documentation, traditionally covers time-spans within a 10- to 1000-year bandwidth. Within that temporal scope, some ‘evolutionary’ cyclical models have been formulated: a cyclical ‘rise and decline’ model for civilizations and empires, and a ‘prestige and neglect’ model for the canonical standing of cultural artefacts. This section concludes by condensing informal points of consensus on human culture into three propositions. Proposition (*a*) summarizes the culture/nature opposition, (*b*) the consensus that human culture is too complex, multicausal and unpredictable for cultural changes to be adequately explained as a simple response to environmental pressures. Proposition (*c*) involves the sentience of the human actors involved and the characteristic quality of cultural self-awareness as an operative force in human culture.

### (a) On the nature/culture distinction

The notion of culture tends to be habitually, albeit not always rigorously, contrasted with its counterpart ‘nature’ (also among Cultural Evolution theorists, cf. [15]). In his *Scienza Nuova*, Vico (1721) saw nature as the physical environment into which humans were born, and as such, while open to human investigation, possibly never wholly knowable (since it exists independently from its being observed or observable by human subjects). In the parlance of the time, Vico saw the investigation of the physical environment as the proper field of ‘(natural) philosophers'. Alongside the philosophy of nature, he placed the analysis of the human experience of life: the investigation of human-made reality, the way humans see, negotiate and express their position in the universe, through practices ultimately based on language and on those language-enabled mental faculties summarized in the Platonic concept of the *λογο*ς.^3^ That logos-analysis Vico called, accordingly, ‘philology’, and the twinning of philosophy and philology was both a trenchant distinction between the physical/empirical and the cultural sciences, and a first conceptualization of what the latter of these dealt with: the human experience of life and the articulations and expressions of that experience. Something, in short, that now we call Culture, which the Oxford English Dictionary, a century or two later, would define as ‘The training, development, and refinement of mind, tastes, and manners; the condition of being thus trained and refined; the intellectual side of civilization’ (OED *s.v*. Culture, 5).

Beyond this commonsensical, fairly rough-and-ready distinction between Culture and Nature, the application of Culture to Evolution, or Evolution to Culture, appears (to a historian's eye) highly varied (cf. also [17,18]). Within the humanities, Culture is linked to the concept of History rather than Evolution. Both deal with the development of systems diachronically (over time), but broadly speaking History would be an idiographic, Evolution a more nomothetic approach to diachronic change.^4^ One commonsensical connotation of the concept of Evolution—rooted in its historical anti-Creationism—is that it is a ‘naturally-’driven, unpremeditated process, taking shape measurably and transgenerationally, whose course is determined by things like ‘natural [i.e. unpremeditated] selection’ and by environmental, external factors. Again, to capture that commonsensical semantic base-line, I turn to the OED: ‘The development or growth […] of anything that may be compared to a living organism […] Also, the rise or origination of anything by natural development, as distinguished from its production by a specific act; ‘growing’ as opposed to ‘being made’’ (OED *s.v.* Evolution, 6).

The tendency for historians to see their preoccupation with diachronic developments as something not quite identical to the notion of ‘evolution’ ties in with their long-established method of collecting their data, not from empirical experimentation (which anyway would run into the classic methodological crux that the past is not repeatable), but from culturally produced documentary evidence, for the analysis of which an important set of procedural validations has been elaborated known as ‘source criticism’ [23–25].

### (b) The cultural causes of cultural change; time-scales and models

There is, then, a robustly established orientation in the humanities to use cultural causes in order to explain cultural effects: what happens in human culture is often caused by the ways in which humans choose to organize their lives, reflecting, in the process, on their lives and on its organization. Culture is often caused by other forms of culture; and the humanities are aware of themselves as also a form of culture (as in fact all forms of science are). The humanities as a scientific discipline are an academically organized way of human self-reflection: human culture looking at human culture. (Hence August Boeckh's definition of philology as *Die Erkenntnis des Erkannten*, ‘the understanding of understanding’ [26]). The extent of available cultural data in standard practice covers the culturally productive, historically documented period of literate, self-representing societies, roughly 3000 BCE to the present, with an archaeological run-up period extending as far back as the beginning of the Holocene. Within this time-frame, culture as an evolving praxis tends to be studied over periods ranging from one or two decades to a few centuries. That median 25–400-year span within the past 1500–5000 years thus sits between the usual temporal span of the social sciences (which is briefer and more concentrated towards the present) and that of archaeologists and palaeontologists.^5^

Starting with Vico himself, some evolutionary models have been formulated on the basis of a long-term comparative study of human cultural productivity (or symbolical self-reproduction). Two are worth mentioning here: the rise-and-fall model and the centre-periphery model.

Vico's idea of a cyclical development of succeeding civilizations in world history saw each civilization emerge from an inarticulate pre-civilized stage into literacy, flourishing, and eventually declining and being overtaken by a different one ([33]; cf. [34]). This emergence-rise-and-fall model, while obviously raising fundamental problems as to its scientific cogency or falsifiability, has had wide-ranging cultural repercussions, prompting a generalized stadial view of human civilization (heralded by Tylor [35]). Speculative and problematic though it is, it still looms large among ethnographers and world-historians, and has become familiar to the general public, mostly as an informal, *a priori* working assumption or ideologeme.

Another tenacious evolutionary model has been developed for the distinction between ‘high’ (canonical) culture and ‘low’ (popular, everyday) culture. Tynjanov [36] and others proposed a systemic model of ‘literary evolution’, where new cultural artefacts vie for symbolical prestige, the more successful ones forming the normative core of the cultural system (its ‘canon’), and then, in a slowly revolving cycle, dropping out of fashion, being relegated again to the system's periphery, and being replaced in the canon by new, successful arrivals emerging from that periphery. Most subsequent cultural canonicity models are refinements of that centre–periphery paradigm. Thus, a multiplicity of coexisting and enmeshing canons-cum-peripheries (in different cultural communities, niches and sub-communities) has been posited (polysystem theory [37]). Also, a two-way dynamics has been factored in to account for the afterlife or rediscovery of outdated cultural artefacts or heirlooms (reception history, memory studies: e.g. [38–40]).

Within the varied palette of the historical humanities, these models for describing cultural history in systemically evolving terms and attempting to formulate a ‘dynamics of cultural history’,^6^ are well established. Their validation does not proceed by way of a formalized experimental procedure where tests are set up to verify or falsify working hypotheses; as I have hinted, the reproducibility of experiments cannot apply in historical research since the past, by definition, is not reproducible. The Popperian criterion of falsifiability is met in two other ways. Any historical analysis before being put to the forum of scholarly opinion needs to be rigorously and source-critically tested against the available data (documentation, existing primary and secondary literature); and its acceptance is always provisional, pending the possible adduction of contradictory data from different sources.^7^ For this reason, evolutionary modelling in the historical sciences has remained tentative and implicit, a matter of ginger consensus rather than explicit assertion, and often more stringently formulated by critics than by proponents. Even so, a few consensual insights from the historical humanities may be summarized as follows, and these may well meet with nods of recognition from evolution researchers in the natural sciences.

### (c) Three propositions on how culture is understood in cultural praxis and the cultural sciences

#### (i) Culture relates to nature as choice relates to conditions

Culture has been defined, in a pithy summary of the culture/nature divide, as ‘anything one could also do differently’ (D Fokkema 1990, personal communication; cf. generally [43]). While natural functions such as eating food, procreation and mortality impose inescapable ‘facts of life’, culture will establish a number of different modalities for negotiating them. Following J.G. Herder's observations regarding the fact that cultural differences between human societies (measured across space or over time) far outweigh physiological differences (cf. [34]; also, from a cultural evolution (CE) perspective, [16]), cultural history works from the prima facie assumption that cultural diversification cannot be wholly physiologically or physically driven. In cultural development, cultural agencies must be involved that must exist and operate (at least partly) autonomously, amounting to more than a mere epiphenomenon or response mechanism.

While cultural practices can, in their differentiation, set different societies apart, they can also be objects of ‘cultural transfer’ [44] and be exchanged between societies. Distribution patterns of culture are never neatly congruent with societal boundaries or demarcations. Inimical, separate populations may share a language and many social patterns (Ireland/England), whereas a single society may have sharply different languages or lifestyles (Scottish Highland/Lowlands; Switzerland). The taxonomy and cladistics of cultural aggregates (dialects/languages) or culturally defined human populations (‘nations’, cf. the tradition from [45] to [46] and [47]) is therefore a vexed question (for much the same reasons as apply to the biological determination of a species-focused taxonomy or cladistics, cf. [48]).

While culture is habitually and universally invoked to identify ‘national’ differences between societies, culture is in fact never a trustworthy proxy for population demarcation. Thus, in archaeology and paleolinguistics, correlations between DNA markers and different cultural traits (burial practices, language) rarely converge into a contradiction-free model (cf. [49]). Culture is not predicated, as a property or characteristic, on a given group; it is, rather, a fluid repertoire of choices that are negotiated within, between and across groups—who at best may or may not derive a subjective sense of collective identity from those choices [47]. Humanities scholars are reluctant to see the self-identification of human groups (as ‘nations’ or ‘races’, prevalent and widely accepted though this is) as something other than an article of belief or ideologeme, changeable over time. Such self-applied group identities are a product of history rather than a condition within which history unfolds.

#### (ii) Cultural developments are nonlinear, multidirectional and multicausal

Culture will as often be marked by the re-emergence of old, dormant practices as by the emergence of new ones. Cultural history develops as a cumulatively expanding repertoire rather than as a successive replacement. Even abandoned cultural practices (e.g. witchcraft in secularized industrialized societies) leave vestigial cultural memories and can be resuscitated; and the old is merely superseded, never wholly abolished.

Examples of culturally highly conservative societies (e.g. China during the Tang and Ming dynasties) are much rarer than examples of cultural change. Since societies need to maintain continuity, culture has a fundamentally preservative, traditionalist component [50]. Nonetheless, change is very widely prevalent in culture. In the shifting power relations within society, Bourdieu [51] has identified an ongoing need for prestige by means of self-distinction (distinguishing oneself from one's lower-status associates) leading to a positive valorization of innovation. Culture accordingly also contains a proclivity to explore beyond the old-established familiar ways. Hence, most cultural systems in recorded history exhibit a bi-stable tension between the need for continuity and the desire for innovation. This tension was invoked by Tynjanov [36] and his ‘Russian Formalist’ school as the driving impetus behind their centre-periphery model of literary evolution; the widely ongoing acceptance of this model is reflected in the core tendency in the humanities to study the relationship between successive historical periods in terms of conceptual binaries such as ‘continuity and change’ or ‘constants and variables'.

These dynamics are generated from within culture itself, not as a response to outside forces; and they do not work in a linearly predictable direction but generate erratic and unpredictable developments. It would be hard, in a historian's vocabulary, to call such dynamics ‘evolution’.

It studying these dynamics, historians face an ongoing challenge to establish which evolving innovations are part of deep paradigmatic transitions and which ones are ephemerally transient and inconsequential in a more long-term context. At what point does a series of hot summers betoken a systemic climate change? Historians have attempted to distinguish between explanatory time-frames of short, medium and long duration [27], without being altogether successful in applying that distinction in practice. There seems to be implicit agreement, however, on an informal momentum-metaphor: the long-term durability of change is equated to the depth of its importance. Short-term innovations are more easily triggered by intra-cultural distinction-desire and as such are liable to be undone or reversed as fashions vacillate (hair length, skirt length, prudery versus libertinism); whereas other, fundamental ones are slow to take hold and almost impossible to reverse. Even here, continuities across deep paradigm shifts are being studied and noted—e.g. the persistence of magical thinking across the societal *Entzauberung der Welt* [52] or the ‘mechanization of our world view’ [53]. And is the modernity process itself, including the cognitive hegemony of scientific rationalism, an irreversible necessity? Can we conceive of anything like de-secularization [54] in other terms than ‘un-boiling an egg’?

Historical change in human societies and human cultures is always marked by the concurrent effects of multiple interacting causes. Historians eschew ‘monocausality’, the idea that a single cause can be identified as the sole agent of historical change. Historical changes can in many instances be derived from a limited number of ‘necessary causes’ (without A being the case, B could not have happened), but to confuse these with ‘sufficient causes’ (once A was the case, B had to happen) imposes from hindsight a teleological determinism. As in other sciences, historians [55–57] reject monocausal modelling as reductive. The well-known ‘urban myth’ that the penchant for hot baths led to the fall of the Roman Empire (through lowering sperm count, or because the plumbing exposed bathers to lead poisoning) has become a proverbial anecdote to illustrate this fallacy. As Danto [55] has argued, explaining ‘changes’ in history from ‘causes’ tends to impose an unwarranted ontological division between these two elements. Upon closer analysis, those ‘causes’ themselves, in human affairs, tend to consist in changes and communicative transitions. Often, changes are not something that happens ‘to’ culture but something that culture makes happen.

#### (iii) The operation of culture involves sentient self-reflection among its participants

Culture is transgenerational. It involves communication between generational cohorts and ensures the survival of its artefacts and practices across them (in what in evolutionary terms is called ‘horizontal’ or ‘oblique’ inheritance). As such, culture establishes a non-physiological or ‘symbolic’ diachronic community for the population, communicatively rather than genetically maintained [58]. As such, culture can be seen as a ‘semiosphere’ existing alongside the human biosphere [59,60]; or it is almost tantamount to what is now called a society's ‘cultural memory’ [61,62].

Culture is a specific function or application of communication rather than communication *per se*. Jakobson's [63] classic taxonomy of linguistic functions lists the communicative function as only one among many. The mere transfer of a piece of information from a sender to a recipient is only a small part of what the communicative act does and effects. Reducing the building of a pyramid, a concert, or a wedding to their communicative function (an afterlife assertion, a public gathering savouring structured soundscapes, a socialized mating ritual) would be, quite literally, a *reductio ad absurdum*. What matters in each of these is precisely what lies beyond their bare communicative functions. A historical case in point is the notorious episode of the 1870 Ems Telegram: Bismarck, in summarizing a diplomatic exchange, suppressed some courtesy circumlocutions which, without altering the substance, changed its flavour, and in so doing soured public opinion and materially hastened the outbreak of the Franco-Prussian War [64,65]. These extra-communicative functions are manifest to the participants involved, and are themselves objects of commentary. Such meta-communication (communicative acts becoming the subject of communicative exchanges) is part and parcel of human interaction. It in turn seems to presuppose socialization, sentience and what is known as ‘cultural literacy’ ([66] and cf. [67]).

Some of these elements may meet with recognition, other with reservations, among scientists working in Cultural Evolution; but all of them, including all of their complexities, are crucial to culture as that concept is used in the Humanities.

## 3. Culture as a recursive system of self-reflexivity

Following on from proposition (iii) in the preceding section—the sentient self-awareness of the actors involved—this section elaborates briefly on the entanglement between culture and meta-culture: the fact that culture is not just an interaction with the environment, but also an interaction with itself. This self-thematization of culture in meta-culture is in fact recursive, and can loop through multiple iterations of reflexivity. The result is a systemic complexity that is characteristic of (though not exclusive to) human cultural systems.

### (a) Meta-culture and cultural self-reflexivity

Central to culture is its capacity to generate meta-culture, ‘cultural reflection or practices conceptualizing cultural reflections or practices'. The humanities and indeed the academic sciences are themselves part of this meta-culture and spend a good part of their time in self-reflexive pursuits (methodological discussions like the present article). This looping recursivity characterizes culture as a complex system, in the technical sense of that word (rather than just a fancy way of saying that ‘it's complicated’). As a complex system, there is no linear correlation in culture between input and output. ‘The’ system when seen as a whole consists of sub-systems, each following their own input–output dynamics as part of the larger whole, and each system is ‘itself’ enmeshed as a subsystem in a larger whole affecting its functionalities. This nesting stack of ‘systems forming part of other meta-systems while themselves containing sub-systems' has no obviously identifiable outer edges, either in an ultimate superimposed container system (short of this being ‘the universe’) or in a fundamental, indivisibly simple building block. The complexity of such an open-ended stacked system also involves multiple, nesting feedback loops and reciprocal transactions within and between sub-systems, generating homeostasis (the ability to self-perpetuate, repair accidental damage and adapt to changing environmental parameters) and (in the case of culture) self-reflexive and symbolical cognition. All this tends to be subsumed under the Luhmann-derived term of autopoiesis ([68] deliberately intended that concept to apply to both physiological and cultural systems). In Luhmann's view [60], society as a system consists not of an aggregate of subjects performing actions, but rather (in line with what I pointed out above) of communicative exchanges, which combine information transfer with a meta-level of reflexive meaning-making about the act of communication. This, as Luhmann puts it, allows communication to be itself perceived as a form of action.

Darwin triumphantly applied a nomothetic evolutionary model to one of the most complex systems of all—the diversification of life-forms on planet Earth. Clearly, the complexity of a system does not preclude its analysis in evolutionary terms. The question is rather, if human culture as a complex system differs from other complex, but ‘natural’ (non-cultural) systems—physiological ones such as the human body, with its organs, cells, organelles and molecular interactions, or inanimate ones such as the weather on planet Earth. I would suggest that there is such a difference and that it is has something to do with the fact that its iterative back-looping self-reflexivity involves what anthropology calls the etic/emic distinction (to be addressed in §4).

In human cognition and interaction, viewed systemically, reflexivity is a key factor: the fact that communicative interactions are not only carriers of information but can become objects of communication, contemplated as actions in their own right. Any question may in response elicit an answer, or the meta-counterquestions ‘What do you mean?’, or ‘Why are you asking me this?’—or all of these at the same time. This reflexivity can loop through multiple feedback iterations. To use a word is not the same as to mention or quote a word, and discussions of the use–mention distinction (e.g. by Sperber & Wilson [69]) can in turn become objects of discussion (as is happening when we discuss Sperber & Wilson's analysis); and so on. Every level of human communication or interaction (what you do, what you think you are doing, how you deal with other people's interpretations of what you are doing, etc.) has its meta-level. The humanities being in a reflective position vis-à-vis the humanity of which they themselves form part, are keenly aware of this fractally nesting structural complexity. Cultural evolutionists, too, as a research community, like any research community, do this all the time.

In their historical dynamics, cultural systems often perform increasing loops through the iteration of reflexivity, with gestures and practices become progressively more ‘aware’ at higher levels of complexity of themselves (etymologically, this is what turns ‘formation’ into ‘in-formation’). Language gives rise to rhetoric and poetics—i.e. reflections on which stylistic figures or formal rules best convey information or narratives, or achieve an aesthetic surplus value. Combat and self-defence are formalized into martial arts, and some of these (like fencing) are performed as sports or displays to acquire social prestige. Nutrition becomes haute cuisine, music becomes a craft (the art of counterpoint and harmony) and then spawns musicology. Traditionalism, once it is consciously experienced as such against the forces of modernity, becomes conservatism [70] and culture itself becomes not only a *habitus* for making sense of the world and structuring its symbolic cohesion (the sort of thing one does without thinking about it [71]), but an object of conscious cultural reflection in its own right (Bourdieu's dualism of *structure structurante* and *structure structurée*), as object of a ‘cultivation of culture’ [72].

## 4. The etic/emic duality: substance and communicative function of cultural signals

Having established the complex, recursively self-observing nature of culture, this section introduces the relationship between what culture consists of (a set of communicative signals) and what functional meaning it carries for its performers. Substance and function of these communicative signals are addressed in the well-known heuristic duality of etic versus emic. In the light of the preceding section, I will argue that the emic function is more than a mere processing of the etic signals as such, but also involves, at recursively nesting meta-levels, meta-reflections on those signals, on the etics and emics of the communicative context of which they form part, and on the act of communication in which they are activated.

The distinction between the etic and emic aspects of human culture has been adopted from the linguistic analysis of human speech-sounds (‘phones’; cf. generally [73,74]). The specialism of *Phonetics* studies phones as physiologically generated acoustic phenomena, in all their variability of how speech organs produce them and what their acoustic qualities are. It was established early on that some phonetic differences are much more important than others, in that they establish differences between words and their separate meanings. In each language, certain phonetic differences are disregarded as being incidental and non-meaningful (allophones, like the end-t in eat or ought), while others carry a meaning-differentiating function: the difference between ‘alive’ and ‘arrive’ or between ‘bed’ and ‘bad’. These latter are called phonemes, and it is from the distinction between phonetics and phonemes that we derive the more generalized differentiation between the –etic and the –emic. The former refers to the physical features constituting a cultural signal, the latter to its generation of meaning as it is being processed. This etic/emic differentiation, it should be stressed, is system-dependent and system-internal, neither universal nor externally determined. The phonemic differentiation between ‘alive’ and ‘arrive’, having no analogue in Japanese phonetics, poses a challenge to Japanese speakers using English; the one between ‘bed’ and ‘bad’, for similar reasons, to Dutch speakers. English speakers, for their part, may fail to notice or pronounce the difference between Russian мат (checkmate) and матъ (mother) with their different end-t's.

As a generalized heuristic duonym, ‘etic’ and ‘emic’ were adopted and widely discussed in anthropology following the work of Kenneth Pike (2nd edn. [75]), by the likes of Lévi-Strauss [76], Harris [77], Olivier de Sardan [78,79] and Sahlins [80]. In anthropologically informed discussions of culture, the *etic* is the ‘hardware’ level of the bare substance of the cultural signals, and the *emic* is the symbolic or social function (meaning) of those signals.

Two important principles are at work here. One is that the emic function of a signal is an intrinsic part of a communicative system, and is conventionally governed by the differentiation structures of that system. At the same time, it is not wholly embedded within its etic substance. The emic meaning of the word 〈verflucht〉 is not determined by what it consists of (a specific string of nine letters in a certain order in the Roman alphabet, with two vowels and six consonants). The meaning is, rather, system-dependent: whether we read it as a Dutch word (meaning ‘the smell of paint’) or an German one (damned); and it would be a meaningless gargle if read as part of a sentence in Basque. At the same time, emic awareness governs our fundamental *a-priori* processing of the etic signal. To be aware whether we understand 〈verflucht〉 as a Dutch or German word affects whether we process its disyllabic structure as *verf-lucht* or *ver-flucht*, its 〈v〉 grapheme as signifying a /v/ or /f/ sound, and the 〈u〉 vowel as in the Dutch name 〈Gullit〉 or in the German name 〈Schubert〉. There is nothing within the word itself to specify all this: not just the understanding, but even the processing of the etic form depend on the meta-information ‘what language are we dealing with here’?^8^

This trivial micro-example illustrates more than that ‘meaning depends on context’; it means that in culture, exchanges are never unidirectional and always involve as much emic sense-processing as etic signal-emission. Establishing the meaning of things (or, iteratively phrased, establishing the meaning of the meaning, as per [81]) is an essential and intrinsic part of human communication, spawning, at the meta-level, the entire philosophical specialism of hermeneutics, which addresses the cognitive operations involved in ‘understanding what is communicated to us'. And this hermeneutical sense-making operates in part on the basis of choosing the systemic context in which to situate the communication. In semiotics (the analysis of how signs convey information), it is understood that for any sign to carry out its function, some ‘collateral’ information is required on the part of the interpretant (cf. [82]); by the same token, language, without the emic function factored in *a priori*, cannot be parsed or processed and becomes indistinguishable from random noise. In the analysis of culture, we cannot strip culture down to its mere etic components. This is why the etic–emic distinction has become such a major issue in anthropological theory. In the Humanities, the paradox that we need to have some understanding of the overall meaning of a text to in order to make sense of its individual parts, while we need to make sense of the individual parts in order to gain an understanding of the overall meaning, is known as the ‘hermeneutic circle’.^9^

Cultural exchanges take place concurrently at three different, interlinked systemic levels: the etic signal as such is sandwiched between the underlying collateral information about its systemic context (situating the message contextually) and the superimposed meta-levels of reflexivity (functionally establishing the meaning of the message and its meaning's meaning). The emic function takes shape as human subjects perform a dynamic parallel processing of these nesting, stacked levels.

## 5. Emic scalarity in cultural systems and its implications for the scientific analysis of culture

This section addresses the issue of systemic scalarity, which is raised by the combination of (a) the recursive complexity of human culture (§3) and (b) the etic–emic dualism of meaning-generation (§4). This section argues that human cultural artefacts are nested in recursive loops of increasing systemic aggregation and complexity, not only as regards their etic components but also as regards the emic functions of these nesting components. These ‘layered’ meanings are operative, and processed, at multiple levels simultaneously. This section also suggests that in the analysis of human cultural artefacts and exchanges, the scalar location of the etic–emic interplay is meaningful. An analysis of high-complexity emic functions (e.g. poetic techniques in a love poem) in terms of etic components embedded at deeply nested systemic levels (e.g. vowels and consonants in the language, hormones in the sex drive) would appear reductive precisely because it ignores this wide scalar gap.

### (a) Stacking: lower and higher aggregation levels of components in cultural artefacts

This interaction between the stacked systemic levels is so tight that it is habitually perceived and performed as a single action. In human practice, the stacked levels that are co-processed are, accordingly, mutually adjacent: so close as to allow instantaneous cross-overs. A brief poetical text (e.g. Gerard Manley Hopkins's sonnet ‘The Windhover’) is commonly read and discussed in terms of the aural effects of its (deliberate) use of assonance involving sounds like /m/ and /d/. But in lengthy texts (Tolstoy's *War and Peace*, or Michelet's *Histoire de la Révolution française*) the relative occurrences of the letters 〈m〉 and 〈d〉 in those texts is situated at such deep-down systemic levels that their emic function is negligible. Such lengthy and complex texts are read and discussed in terms of their large-scale features: narrative themes and techniques, discursive arguments and rhetoric (which at a more fine-grained level involve the tropes and stylistic choices (which at an even more fine-grained level involve the arrangement of statements and events (which involve choices of words (which happen to contain letters like 〈m〉 and 〈d〉)))). Scalarity, in other words, matters.

Texts come to us as structures with multiple nesting, stacked levels of increasingly complex aggregation and organized by choices and effects at micro-, meso- or macro-level; and at each of these different levels emic signalling functions have their role to play alongside their etic substance; each of them requires collateral, externally sourced information to situate them and each of them can invite or generate meta-reflection. And each emic function is, I believe, level-specific.

I am merely and diffidently suggesting, as a hypothesis to consider, that this stacked multi-level interplay between etic features and emic functions is particular to human-constructed cultural systems. But I am confident that the systemic characteristics identified here single out the humanities, rather than the empirical sciences, as the best-equipped discipline to investigate culture's twinning of complexity and multiply embedded emic functions. Culture cannot, I feel, be studied adequately on the basis of a mechanical registration of its etic operations, as if it were a set of thermal currents in a volcano, or molecules arranging themselves into a polymer or protein. This, I think, is what Dilthey [84] meant when he made his classic distinction between *erklären* and *verstehen*, the former (explaining) being a type of comprehension proper to the natural sciences, the latter (understanding) specific to the humanities, the former concentrating on the question ‘how’, the latter gravitating to the question ‘why’.

### (b) Low-level etic elements have a diminishing emic function at higher stack levels

I draw another inference. The nesting, stacked and scalar logic of culture, which at each level involves emic functions in the processing of etic signalling, makes it problematic to discussing highly structured cultural phenomena or artefacts by processing the etics of their component sub-systems at deeply subordinate structural levels. It might make sense to analyse the letters (or even ink pixels) as components of *War and Peace* for mechanical purposes (such as, for instance, optical character recognition algorithms), but this approach would not amount to literary criticism since the literary features of the identical text might be fruitfully discussed on the basis of wholly differently composed letters or typesettings, or a handwritten copy, or even a translated version. Much as we do not discuss novels in terms of the letters or ink pixels that make up books, so too Michelin stars are awarded to restaurants, not on the basis of what nutrients their dishes contain, but for their highly structured presentation of dishes (that are prepared in complex procedures (making use of ingredients (containing those nutrients))).

Much as it is trivial to define the human body as consisting of protons, neutrons and electrons, so too it is question-begging to define developments in modern/Western musical culture not on how people chose to resolve diminished-ninth chords or emically perceived dissonants (cf. [85]), but at the particle-level of sound-frequencies. Analysing low-level etic components of highly structured cultural artefacts may, of course, have its uses—e.g. when discussing the algorithms of optical character recognition, or the health hazards of *haute cuisine*, or Digital Rights Markers in audio datasets. But the way in which the artefact as a whole is processed, its meaning and historical presence in the culture (e.g. the social status of champagne or of a law text, or the stylistic development of jazz music) is not ‘explained’ thereby. While we can now process Big Data, i.e. the quantitative plethora of such low-level components, that does not yet take into account the complex structures from which we extract those Big Data. Seeing culture as Big Data reduces it to Flat Data. We no longer discuss novels, dinner menus or musical compositions, but merely printed matter, food or sustained concatenations of structured noise.

## 6. Conclusion (know what you're saying when you say ‘culture’)

‘Culture’ is not a neologism or a *terminus technicus*. It is embedded in the human experience of the world rather than an analytical concept applied to operations in the world. It is both older and more specific in its *Begriffsgeschichte* than more technical and recently coined words like ‘behaviour’ or ‘communication’. When applying the word ‘culture’ to ‘evolution’, this historically accreted specificity of meaning should, I feel, be factored into the analysis, which accordingly should take account of its inherent complexity and emic, meaning-making functions.

In many cases, Cultural Evolution studies non-genetic information transfers, even across generations, that do not involve such complexities and emic functions. In those cases, I feel, it would be more precise to speak of *behavioural* or *communicative,* rather than ‘cultural’ processes. Many of these processes undoubtedly affect natural selection pressures on coevolutionary processes, without being either strictly physiological or, strictly speaking, ‘cultural’ (cf. [16]). In such cases, much confusion would be avoided—and a greater degree of species-neutral clarity would be achieved—if one were to speak of ‘Communicative Evolution’ or ‘Behavioural Evolution’ rather than ‘Cultural Evolution’. To describe non-physiological, non-emic and non-human forms of information transfer and information-maintenance as ‘cultural’ may be rhetorically attractive but is factually, to my mind, inappropriate. It repeats the rhetorical wink, signalled at the outset of this essay, to mechanize humanity while imputing human-style (emically complex) emotionality to low-emic or mechanical operations. ‘Spitefulness in octopuses' (as per the publicity around [86]), ‘culture among fruit flies’ [87], or even ‘selfishness in genes': such phrasings are, strictly speaking, an oxymoron or over-extended metaphor, a turn of phrase that tickles the reader but fuzzes the issue.

That being said, there are exciting new vistas being opened by the application of very powerful empirical methods and data analysis, also when applied to human culture; and the use of iterative modelling does indeed raise the potential of seeing cultural patterns evolve, in the true sense of the words ‘culture’ and ‘evolution’. By way of example I point out a few.

(1) Improved recording devices make it possible to study the etics and emics of human interaction objectively at a very fine-grained level. The registration of pupil dilation or (in infants) pacifier-sucking when experiencing verbal stimuli has helped our understanding of ‘deep’, physical–habitual semantic and phonemic processes considerably. Computer modelling such deep-structural processes across repeated iterations (as a proxy for human generations) may, in well-chosen experimental cases, be equally useful for evolutionary theories of language.
(2) Digitization has exponentially increased the production of cultural signals (from tweets to Internet memes) and exponentially enlarged the corpus of analysable data. These very large and often ephemeral cultural corpuses (Big Data in the true sense) would traditionally (manually) have been impervious to data capture or analysis, but can now become fertile objects for computerized searches and analytical modellings.
(3) Computer modelling has also vastly extended our power to analyse complex systems in all their nonlinearity and nesting structuration. That holds out very exciting prospects also for the modelling of cultural dynamics; and for all the caveats sounded in the foregoing pages I would by no means wish to exclude the possibility that these cultural dynamics do exhibit ‘evolutionary’ patterns over time. That burden of proof demands more than a single-instance, simplified proof of concept; but the perspectives are enticing.

## Footnotes

1 Recent fresh reflections on the distinctness and mutual complementarity of History and Anthropology were started by Berlioz *et al*. [7]; for a relatively recent stock-taking, see [8].

2 E.g. [11] asserting ‘We Are Our Brain’ (and cf. the critique by Noë [12]), or [13] using the phrase ‘Free will doesn't exist’ as a popularization of serious work like [14].

3 As readers will realize, this culturally established, anthropological meaning of culture is much wider than the evolutionary exigencies of ‘niche construction’ to which some biologists [16] would subordinate it.

4 I take the heuristic distinction between idiographic and nomothetic sciences (the former striving to account for singularities, the latter striving to subsume and generalize phenomena into regularities) from Windelband's classic *Geschichte und Naturwissenschaft* [19], where it maps fairly neatly onto the Vicoesque distinction between the historical humanities and the exact sciences; cf. also Thomae [20]. For the notion that the humanities, idiographic as they are, are nonetheless more than a mere descriptive exercise and seek to generalize their insights, see [21]. Kamlah's critique of Windelband, however [22] seems overstated: the fact that the idiographic/nomothetic distinction is not in all respects tenable does not mean that it is wholly untenable.

5 Such ‘deep’ or ‘big’ histories, given their propensity to deal with multimillennial periods, tend to rely on existing research and collate selections of existing data, re-narrating or synthesizing these. Some social and economic historians have addressed large-scale, long-term processes, which would often deal with the competitive rise and fall of entire societies, often in interaction with large-scale ecological shifts. These range from the idea of *longue durée* processes and World System theory [27,28] to more recent developments such as Macrohistory ([29], with popular best-sellers such as Diamond [30] and Harari [31]). Initiatives such as Cliodynamics [32], largely web-based, attempt to use computer modelling for explaining long-term and/or large-scale historical change. But such methodological and theoretical debate as this line of research engenders does not amount to a two-way conduit between the theory or history or of the humanities and that of evolutionary science, and some scientists in the Cultural Evolution field are on record as dismissing such long-term quasi-evolutionary histories as ‘adaptive storytelling’ (F Weissing 2020, personal communication). Generally on time-scales in CE research: [3].

6 Witness the programme on *Cultural Dynamics: Inheritance and Identity* under the auspices of HERA, a consortium of funding agencies for the humanities in the European Research Area, 2009–2012: http://heranet.info/projects/hera-i-cultural-dynamics-inheritance-and-identity/. For an attempt to model cultural dynamics from an anthropological starting point, see [41].

7 For this reason, Rigney [42] sees the relationship between successive historians as necessarily ‘agonistic’.

8 Cf. [76] on the emic, culturally determined feedback on how human vision processes light in different wavelengths (colours).

9 Cf. [80]. Anthropologists have come to the conclusion that the desire to give a clean, unbiased and ‘objective’ description of cultural signs, unburdened by interpretative biases and crisply focused on the pure etic components of culture, is a chimera, like sawing a magnet in half in order to study only its positive, not its negative pole. The urge was born from the understandable desire to winnow the observer's own cultural presuppositions and emic sense-making out of the observation, but it is now realized that emic meaning-making functions are part and parcel of culture, even at the most fine-grained and fundamental level, not some external flavouring addition to it. For the ‘Hermeneutic Circle’, see [83].

## Method

Methodological scrutiny of printed sources and existing literature.

## Data accessibility

This article has no additional data.
